# Oral immunization with a novel attenuated *Salmonella* Gallinarum encoding infectious bronchitis virus spike protein induces protective immune responses against fowl typhoid and infectious bronchitis in chickens

**DOI:** 10.1186/s13567-018-0588-9

**Published:** 2018-09-12

**Authors:** Irshad Ahmed Hajam, Jehyoung Kim, John Hwa Lee

**Affiliations:** College of Veterinary Medicine, Chonbuk National University, Iksan, 54596 Republic of Korea

## Abstract

Fowl typhoid (FT), a septicemic disease caused by *Salmonella* Gallinarum (SG), and infectious bronchitis (IB) are two economically important avian diseases that affect poultry industry worldwide. Herein, we exploited a live attenuated SG mutant, JOL967, to deliver spike (S) protein 1 of IB virus (V) to elicit protective immunity against both FT and IB in chickens. The codon optimized S1 nucleotide sequence was cloned in-frame into a prokaryotic constitutive expression vector, pJHL65. Subsequently, empty pJHL65 or recombinant pJHL65-S1 plasmid was electroporated into JOL967 and the resultant clones were designated as JOL2068 and JOL2077, respectively. Our results demonstrated that the chickens vaccinated once orally with JOL2077 elicited significantly (*p* < 0.05) higher IBV-specific humoral and cell-mediated immunity compared to JOL2068 and PBS control groups. Consequently, on challenge with the virulent IBV strain at 28^th^ day post-vaccination, JOL2077 vaccinated birds displayed significantly (*p* < 0.05) lower inflammatory lesions in virus-targeted tissues compared to control groups. Furthermore, 33.3% (2 of 6) of birds vaccinated with JOL2077 vaccine had shown virus recovery from tracheal tissues compared to 100% (6 of 6) recovery obtained in both the control groups. Against wild-type SG lethal challenge, both JOL2077 and JOL2068 vaccinated groups exhibited only 10% mortality compared to 80% mortality observed in PBS control group. In conclusion, we show that JOL2077 can induce efficient IBV- and carrier-specific protective immunity and can act as a bivalent vaccine against FT and IB. Further studies are warranted to investigate the potential of JOL2077 vaccine in broiler and young layer birds.

**Electronic supplementary material:**

The online version of this article (10.1186/s13567-018-0588-9) contains supplementary material, which is available to authorized users.

## Introduction

Infectious bronchitis virus (IBV), one of the prototype viruses of the *Coronaviridae* family, is an enveloped, single-stranded, positive-sense RNA virus that causes significant economic losses in poultry industry worldwide [1, 2]. In chickens, IBV causes an acute highly contagious disease which affects the respiratory tract, gut, kidneys, bursa and reproductive systems, and the disease is characterized by 50–100% morbidity and 0–25% mortality rates in affected flocks [3, 4]. The viral genome encodes four major structural proteins, namely membrane protein (M), nucleocapsid protein (N), spike protein (S) and envelope protein (E), that facilitate host entry and virus assembly [5]. The S protein is post-translationally cleaved by host cell serine proteases to form two subunits, S1 and S2, and this cleavage plays an important role in viral infectivity [6]. The S1 subunit binds to host cell receptors and is the main target for induction of serotype-specific neutralizing and hemagglutination-inhibiting antibodies [7, 8], and it is sufficient to induce protective immune responses [9]. To prevent economic losses resulted by IB, live attenuated and inactivated oil adjuvanted IBV vaccines have been employed in the field [9]; however, these types of vaccines are associated with some limitations. Inactivated IBV vaccines are safe, but relatively costlier and less effective than live attenuated vaccines, while use of live attenuated vaccines may result in the emergence of novel variants of the virus [10, 11]. Furthermore, the existence of more than 20 IBV serotypes with little cross protective immunity among vaccine strains results in poor efficacy of the currently available vaccines. The introduction and development of an effective and safe vaccine against each IBV serotype is not an economically viable option for the poultry industry. Therefore, novel approaches should be devised that are not only egg independent and cost-effective, but easy to amplify and can provide efficient protection against the circulating IBV strains.

Identification and characterization of regional and most circulating serotypes plays an important role in the development of a successful vaccination program. Over the past decade, nephropathogenic IBV variants have been continuously evolved in South Korea via accumulated point mutations and by recombination with other existing strains [12]. Thus, emphasizing the need for the development of efficient and effective vaccination strategies which respond to the circulating field strains in a very short notice. In accordance with this notion, the present study exploited *Salmonella* Gallinarum (SG) to deliver immunogenic S1 protein (20–263 amino acid residues) of most emerging and circulating nephropathogenic strain of IBV in South Korea. SG is an intracellular Gram-negative bacterium that causes fowl typhoid (FT) in domestic birds, primarily chickens, and rarely causes food poisoning in humans [13]. In chickens, FT is characterized by severe inflammation and acute mortality that causes significant economic losses in poultry industry worldwide [13–15]. The disease has worldwide distribution and is endemic in many parts of the world. Besides farm hygiene and biosecurity measures, the control and eradication of FT can be effectively achieved by vaccination with live attenuated and inactivated killed vaccines. We previously have developed an attenuated SG vaccine strain (JOL916) that effectively controls FT infection in chickens [16]. Exploiting live attenuated *Salmonella* system to deliver IBV S1 immunogenic protein is a highly economical vaccination strategy that allows for a quick response to deployment of vaccines, as it circumvents the need for a constant supply of specific-pathogen-free (SPF) embryonated eggs. Furthermore, *Salmonella*-based vaccination strategy induces efficient antigen-specific humoral and cell-mediated immunity [17–19], and these vaccines have already been approved for use in poultry [20]. Herein, we show that attenuated SG mutant delivering immunogenic S1 protein elicited efficient IBV-specific humoral and cell-mediated immune (CMI) responses and conferred significant protection against a virulent IBV challenge. We also demonstrate that attenuated SG delivering S1 protein protected chickens against a lethal wild-type SG challenge.

## Materials and methods

### Bacterial strains, plasmids and virus

The bacterial strains and plasmids used in this study are listed in Table 1. All the bacterial strains were grown in Luria–Bertani (LB) broth at 37 °C. IBV strain was purchased from Korea Veterinary Culture Collection (KVCC-VR1100015; South Korea) and propagated in the allantoic cavities of 9- to 10-day-old SPF embryonated chicken eggs. The 50% egg infective dose (EID)_50_ was calculated as described previously [21].

**Table 1:** **Bacterial strains and plasmids used in this study**

Strains/plasmids	Description	References
Strains		
Χ232	*E. coli* Δ*asd* strain used for cloning of genes into *asd*^+^ plasmid	[28]
BL21(DE3)pLysS	F^−^, *omp*T, *hsd*S_B_ (r_B_^−^, m_B_^−^), *dcm, gal*, λ (DE3), pLysS, Cm^r^	Promega, USA
JOL967	*∆lon, ∆cpxR and ∆asd* mutant *of* SG	[28]
JOL916	*∆lon* and *∆cpxR mutant of* SG	[28]
JOL2068	JOL967 with pJHL65 plasmid	This study
JOL2077	JOL967 with pJHL65-S1 plasmid	This study
JOL1992	BL21(DE3)pLysS with pET28a-S1 plasmid	This study
Plasmids		
pJHL65	An *asd*^+^ vector, pBR ori, β-lactamase signal sequence-based periplasmic secretion plasmid, 6xHis, high copy number	
pJHL65-S1	pJHL65 carrying IBV S1 gene sequence	This study
pET28a (+)	IPTG-inducible expression vector; Kan^r^	Novagen, USA
pET28a-S1	pET28a plasmid carrying S1 gene	This study

### Construction of an attenuated SG mutant delivering IBV spike protein 1

The partial immunogenic S1 amino acid sequence_20–263_ of most emerging nephropathogenic IBV Korean strain (Accession No. ADQ01077.1) was chosen, in this study, based on the presence of major neutralizing B and T cell epitopes that are well-known to elicit complete protection against IBV infection in vaccinated chickens [22–24]. The S1 nucleotide sequence was codon optimized for efficient expression in SG. The optimized gene sequence was chemically synthesized (Bionee, Korea) and then built into the pJHL65 plasmid, an *asd*+ constitutive expression vector, and propagated in an *asd* mutated *Escherichia coli* strain as described previously [25]. The S1 gene sequence was cloned in frame downstream to the beta-lactamase signal sequence (*bla SS*) of pJHL65 vector, so that the intended protein would get secreted out of the bacteria [26]. The recombinant plasmid, pJHL65-S1, was subsequently transformed into an attenuated auxotrophic mutant of SG strain, JOL967, and the resultant clone was designated as JOL2077. The JOL967 strain was constructed by the deletion of the *lon*, *cpxR*, and *asd* genes from the wild-type SG strain, JOL394 isolate, using allelic exchange method as described and reported elsewhere [16], and used as the delivery vehicle for the S1 protein. To produce the coating antigen for determination of IBV-specific antibody responses, the S1 gene was cloned into pET28a (+) expression vector (Novagen, San Diego, USA) that was subsequently transformed into *E. coli* BL21 (DE3) pLysS strain (Novagen, USA) for protein expression. The expressed S1 protein in both *S. typhimurium* and *E. coli* was confirmed by Western blot analysis using polyclonal infectious bronchitis virus antibody (#ab31671, Abcam). The *E. coli* expressed S1 protein was purified by Ni–NTA affinity column chromatography and dialysed against PBS (three washes). Purified protein was quantified by a Bradford assay [27], filtered, and stored at −20 °C until further use.

### Immunization and challenge studies

All animal experimentation work was approved by the Chonbuk National University Animal Ethics Committee (CBNU2015-00085) and the chicken experiment was carried out according to the guidelines of the Korean Council on Animal Care. One-day-old female layer chickens (Corporation of Join hatchery, Republic of Korea) were maintained under standard conditions and provided antibiotic-free food and water ad libitum. Four weeks later, the chickens were randomly divided into three groups (*n* = 15) and vaccinated once orally with sterile phosphate buffered saline (PBS, 100 µL in each bird), JOL2068 (SG carrying an empty pJHL65 vector) or JOL2077 (SG-pJHL65-S1). The 10^9^ colony forming units (CFU) of JOL2068 or JOL2077 were used for inoculation in each bird. Blood was drawn from jugular vein of six randomly vaccinated chickens on day 28 post-vaccination and serum samples were subsequently isolated for IgG analysis. To assess mucosal secretory (s) IgA responses, five chickens on day 28 post-vaccination were sacrificed from each group and intestinal wash samples were collected in PBS (pH 7.2) containing antibiotics and protease inhibitor cocktail (Sigma Aldrich).

Four weeks post-vaccination, all the vaccinated chickens were intranasally challenged with a virulent dose of IBV (10^4^ EID_50_) and 6 days later post-challenge, the tracheal tissues (*n* = 6) were aseptically processed and inoculated into 15–16 day old chicken embryos to isolate the challenged virus as described previously [21]. The clinical protection against IBV was assessed based on the death and/or stunting of embryos. For histopathological analysis of trachea, lungs, kidneys and bursa, each tissue from sacrificed birds (*n* = 6) was aseptically collected on day 6 post-challenge and fixed with 10% formalin, embedded in paraffin and cut into 5 μm thin sections, which were subsequently processed as previously described [18]. The hematoxylin and eosin (H&E) stained sections were examined by light microscopy (Axio Imager 2, Zeiss, Germany) and digital imaging software (Axio Vision, Zeiss, Germany). We also separately maintained three groups (*n* = 10) vaccinated once orally with JOL2068, JOL2077 or sterile PBS, and 4 weeks later, all the birds were challenged orally with 100 µL of a suspension containing 1.6 × 10^6^ CFU of a wild-type SG (JOL394 strain) as described previously [28]. The protective efficacy of the SG-S1 based vaccine (JOL2077) against JOL394 was evaluated in the context of the mortality of the challenged birds, which was monitored daily for 12 consecutive days. At the end of the observation period post-challenge, the surviving birds were sacrificed to determine the bacterial load in liver and spleen as previously described [28].

### Systemic IgG and mucosal IgA antigen-specific humoral responses

The systemic IBV and SG-ompA specific IgG and mucosal IgA responses were analysed in vaccinated sera and in intestinal wash samples, respectively, by an indirect ELISA [28]. Purified S1 protein and ompA antigen (each 250 ng/well) were used as coating antigens in an indirect ELISA to determine the IBV and the SG specific humoral responses, respectively.

### IBV-specific cellular immune responses

The IBV-specific cell-mediated immunity elicited by vaccination was evaluated by a lymphocyte proliferation test. Four weeks post-vaccination, in vitro proliferative capacity of vaccinated peripheral blood mononuclear cells (PBMCs) (1 × 10^6^, *n* = 6) in response to the recall S1 protein was determined by a MTT [3-(4,5-Dimethylthiazol-2-yl)-2,5-diphenyltetrazolium bromide] based assay [18]. The PBMCs were isolated using Histopaque density gradient as previously described [18]. The proliferative capacity of vaccinated PBMCs in response to recall S1 antigen was further analyzed by flow cytometry. To this end, PBMCs (1 × 10^6^, *n* = 6) isolated from vaccinated birds on day 28 post-vaccination were stimulated with S1 antigen (10 µg/mL) for 48 h and then S1-specific CD4+ and CD8+ T cell responses were analysed by flow cytometry as previously described [28].

For analysis of Th1 (IFN-γ) and Th2 (IL-4 and IL-10) cytokine responses, PBMCs (1 × 10^6^, *n* = 6) were isolated from vaccinated and control birds and restimulated with S1 protein (10 µg/mL) in vitro for 24 h incubation at 37 °C in 5% CO_2_. Then total RNA isolated from stimulated cells was analysed for IFN-γ, IL-4 and IL-10 mRNA transcription levels by qRT-PCR assay as previously described [28].

### Statistical analysis

All the obtained data was analysed using GraphPad prism 6.00 program (San Diego, CA, USA). Statistical significance was determined by one-way ANOVA (with Tukey’s multiple comparisons tests). A non-parametric Chi square test was used to analyze significant differences in mortality of birds following SG challenge. Data are represented as mean ± standard deviation. *p* values of < 0.05 were considered statistically significant.

## Results

### Attenuated SG mutant efficiently expressed and secreted IBV S1 protein

To construct the SG-based IB vaccine, we predicted highly antigenic epitopes of IBV S1 protein using BepiPred 1.0 Server program based on the protein characteristics such as structural domains, hydrophilicity residues, antigenicity and surface probability (Additional file 1) [29]. To direct the expressed protein to the periplasmic space, the S1 gene was cloned in frame downstream to the *bla SS* of the pJHL65 vector. The insertion of S1 into pJHL65 vector was confirmed by digestion of the positive clones with *Eco*R1 and *Hin*dIII to release a fragment of 738 bp. Subsequently, the pJHL65-S1 gene construct was electroporated into JOL967 strain and the resultant clone was designated as JOL2077. Western blot analysis revealed that JOL2077 strain efficiently expressed and secreted S1 protein in the culture supernatant of cultured JOL2077 bacteria (Additional file 2). The S1 protein was biologically active as evidenced by the reactivity with polyclonal IBV-specific antibody and by the induction of the antigen-specific immune responses in vaccinated chickens as observed in this study.

### Orally administered JOL2077 vaccine induces efficient S1-specific systemic and mucosal antibody responses

**Figure 1:**
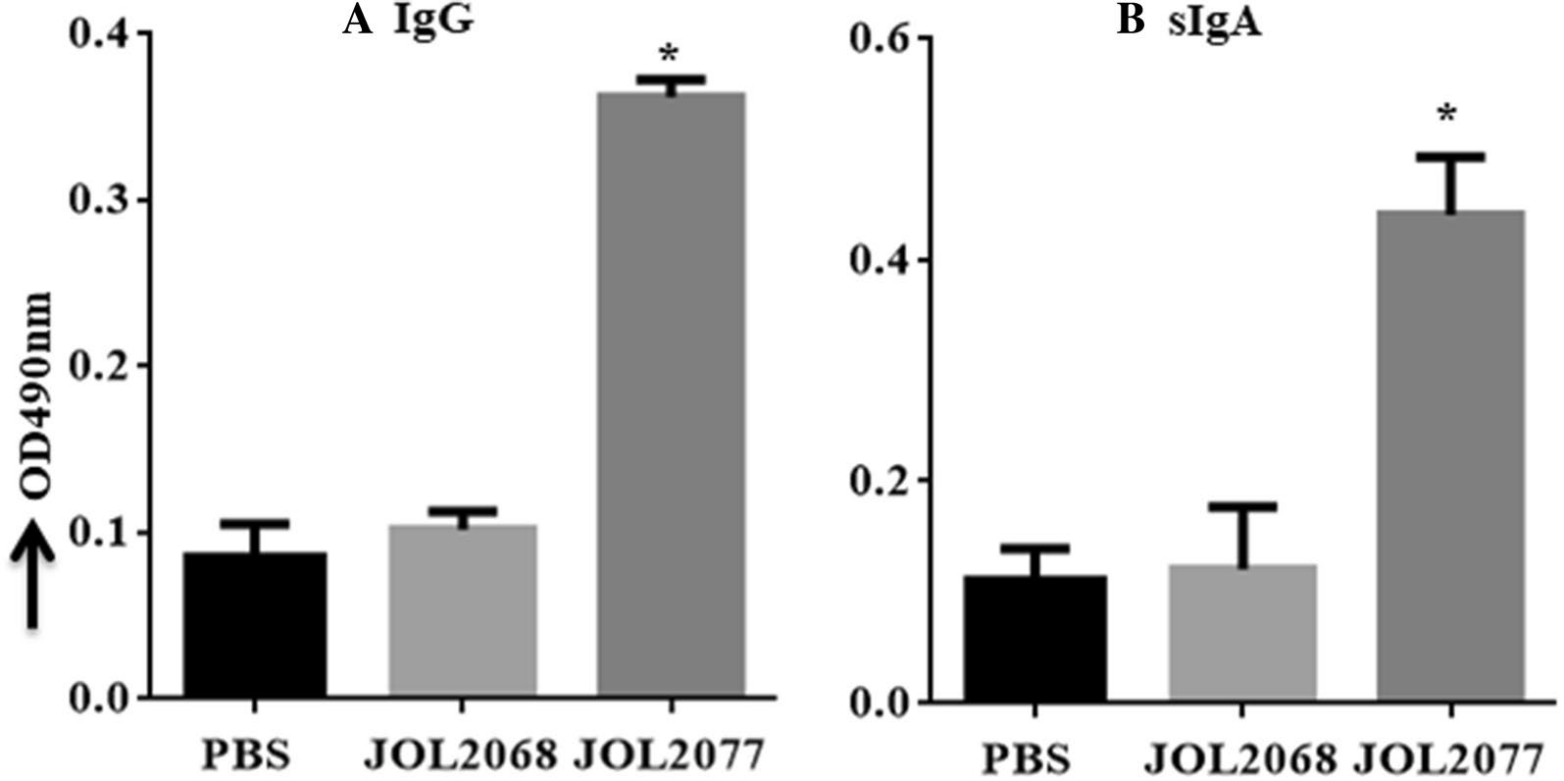
**IBV-specific systemic IgG and mucosal IgA responses.** Chickens (*N* = 15) were vaccinated with sterile PBS, JOL2068 or JOL2077, and 28 days later serum and intestinal wash samples were analysed for S1-specific IgG and IgA responses, respectively, by an indirect ELISA. **A** Serum IgG. **B** Intestinal sIgA responses. Each data point represents mean ± SD of six chickens. *p < 0.05.

To investigate the effect of vaccination on the systemic IgG and the mucosal sIgA antibody responses, we orally vaccinated chickens with PBS, JOL2068 or JOL2077, and 28 days later IgG in serum and sIgA in intestinal wash samples were measured by an indirect ELISA (Figure 1). Our results demonstrated that the chickens inoculated once orally with JOL2077 elicited significantly (*p* < 0.05) higher both IgG and sIgA responses compared to the chickens that received either PBS or JOL2068 strain (Figure 1). The S1-specific IgG (Figure 1A) and sIgA (Figure 1B) responses were 3.6 and 3 folds, respectively, higher than JOL2068 control group. Our data thus clearly indicate that attenuated SG delivering S1 protein is capable of inducing antigen-specific systemic and mucosal humoral immune responses.

### Orally administered JOL2077 vaccine elicits efficient IBV-specific cellular immunity

**Figure 2:**
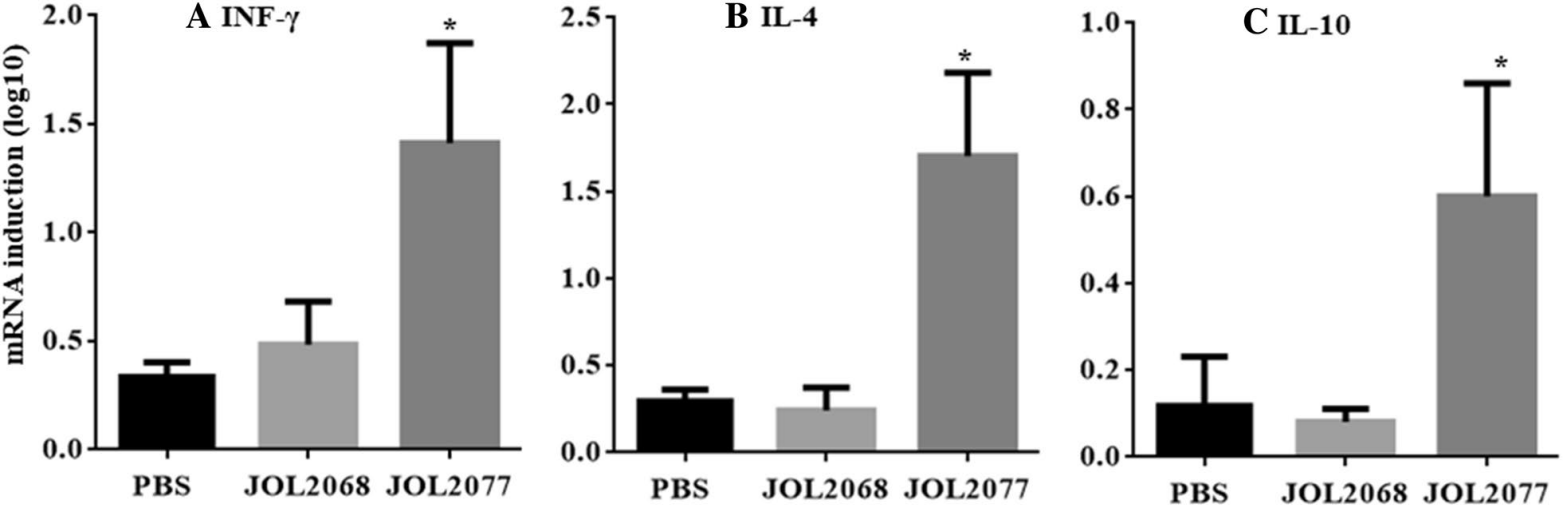
**Analysis of cytokine gene expressions in PBMCs after stimulation with S1 antigen.** Chickens (*N* = 15) were vaccinated with sterile PBS, JOL2068 or JOL2077, and PBMCs from vaccinated chickens after 28 days post-vaccination were stimulated with purified S1 antigen for 24 h and then analysed for induction of IFN-γ, IL-4 and IL-10 mRNA transcription levels by qRT-PCR assay. Results are expressed as log10 fold induction in mRNA transcription levels of stimulated PBMCs from vaccinated chickens compared to the unstimulated naïve PBMCs. Gene expressions were normalized to GAPDH and mRNA levels of naive cells were used as the calibrator. Data presented are mean ± SD of six chickens per group. *p < 0.05.

To assess the effect of vaccination on the IBV-specific CMI responses, we measured mRNA levels of IFN-γ, IL-4 and IL-10 cytokines in vaccinated PBMCs restimulated with S1 protein in vitro for 24 h (Figure 2). Our results demonstrated that the chickens vaccinated with JOL2077 vaccine induced significantly (*p* < 0.05) higher mRNA levels of both Th1-type (IFN-γ) and Th2-type (IL-4 and IL-10) cytokines compared to the JOL2068 and the PBS control groups (Figure 2). The IFN-γ, IL-4 and IL-10 cytokines were 3.5, 5 and 4 folds, respectively, higher in JOL2077 vaccinated chickens compared to the JOL2068 control chickens, which showed non-significant and comparable levels to that of the PBS control chickens. Our data further revealed that mRNA levels of Th1 and Th2 cytokine levels were almost comparable, suggesting that vaccination with JOL2077 vaccine elicited mixed-type of immunity.

**Figure 3:**
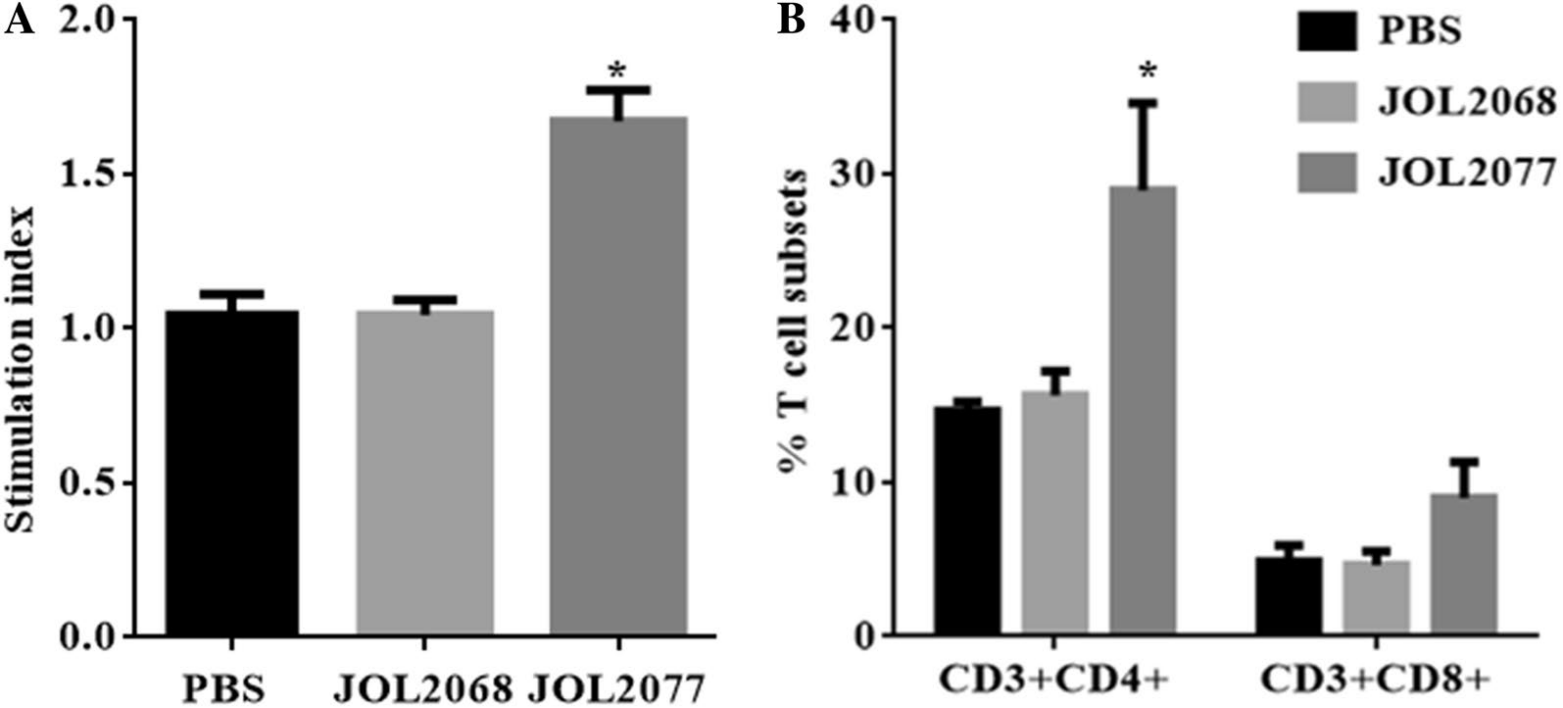
**IBV-specific cellular immune responses. A** In vitro proliferations of lymphocytes from vaccinated chickens in response to the recall S1 antigen. Results are expressed as stimulation indices, defined as proliferation in response to recall antigen relative to the mock stimulated cells. Each data points represent mean ± SD of six chickens per group. **B** The CD3+CD4+ and CD3+CD8+ T-cell subsets in vaccinated and control chickens are expressed as a percentage of the gated cells. The data are presented as the mean ± S.D of six chickens. *p < 0.05.

Next, we measured T cell proliferative responses by a MTT-based assay after restimulation of vaccinated and control PBMCs with S1 protein in vitro for 72 h. The proliferative responses were significantly (*p* < 0.05) higher in chickens that received JOL2077 vaccine compared to the JOL2068 and PBS treated groups (Figure 3A). The lymphoproliferative responses were consistent with the results of CD4+ and CD8+ T cell responses measured by flow cytometry after restimulation of vaccinated PBMCs with S1 protein in vitro for 48 h (Additional file 3). Our results indicated that JOL2077 vaccinated chickens elicited significantly (*p* < 0.05) higher IBV-specific CD4+ T cell responses compared to the JOL2068 and the PBS control groups (Figure 3B). Although CD8+ T cell responses were higher in JOL2077 vaccinated chickens, but the responses were non-significant compared to the PBS and the JOL2068 control groups (Figure 3B). Thus, our results clearly demonstrate that oral inoculation of SG delivering S1 protein is capable of eliciting both IBV-specific humoral and CMI responses.

### Orally administered JOL2077 provides protection against virulent IBV challenge

To evaluate whether SG-based S1 vaccine could elicit immunoprotection against IB, we intranasally challenged all the vaccinated and the control chickens with a virulent dose of 10^4^ EID_50_ IBV at 4 weeks post-vaccination. No mortality was observed in any of the chicken groups, but chickens in control groups showed depression, huddling and anorexia, and even diarrhea was observed in some birds. In contrast, JOL2077 vaccinated birds appeared normal and free from any disease symptoms. The protective efficacy of the JOL2077 vaccine was further analyzed by isolating the virus from tracheal tissues on day 6 post-challenge. The virus was recovered from 33.3% (2/6) of birds vaccinated with JOL2077 vaccine compared to the 100% (6/6) recovery obtained in both JOL2068 and PBS control groups (Table 2). These results clearly indicate that vaccination with JOL2077 vaccine had efficiently contained IBV replication inside the host tissues and thus protection from the clinical disease.

**Table 2:** **Protective efficacy of orally administered SG-based S1 vaccine (JOL2077) in brown nick layer chickens**

Groups	Recovery of virus from trachea No. positive/no. tested	% protection^a^
PBS	6/6	0
JOL2068	6/6	0
JOL2077	2/6	66.7

Chickens were challenged with virulent IBV strain nasally at 4 weeks post-vaccination. The challenged virus was isolated from the trachea at 6^th^ day post-challenge and the immunoprotection efficacy was assayed.

^a^Percent protection was determined as the number of positive chickens/total number of chickens.

**Figure 4:**
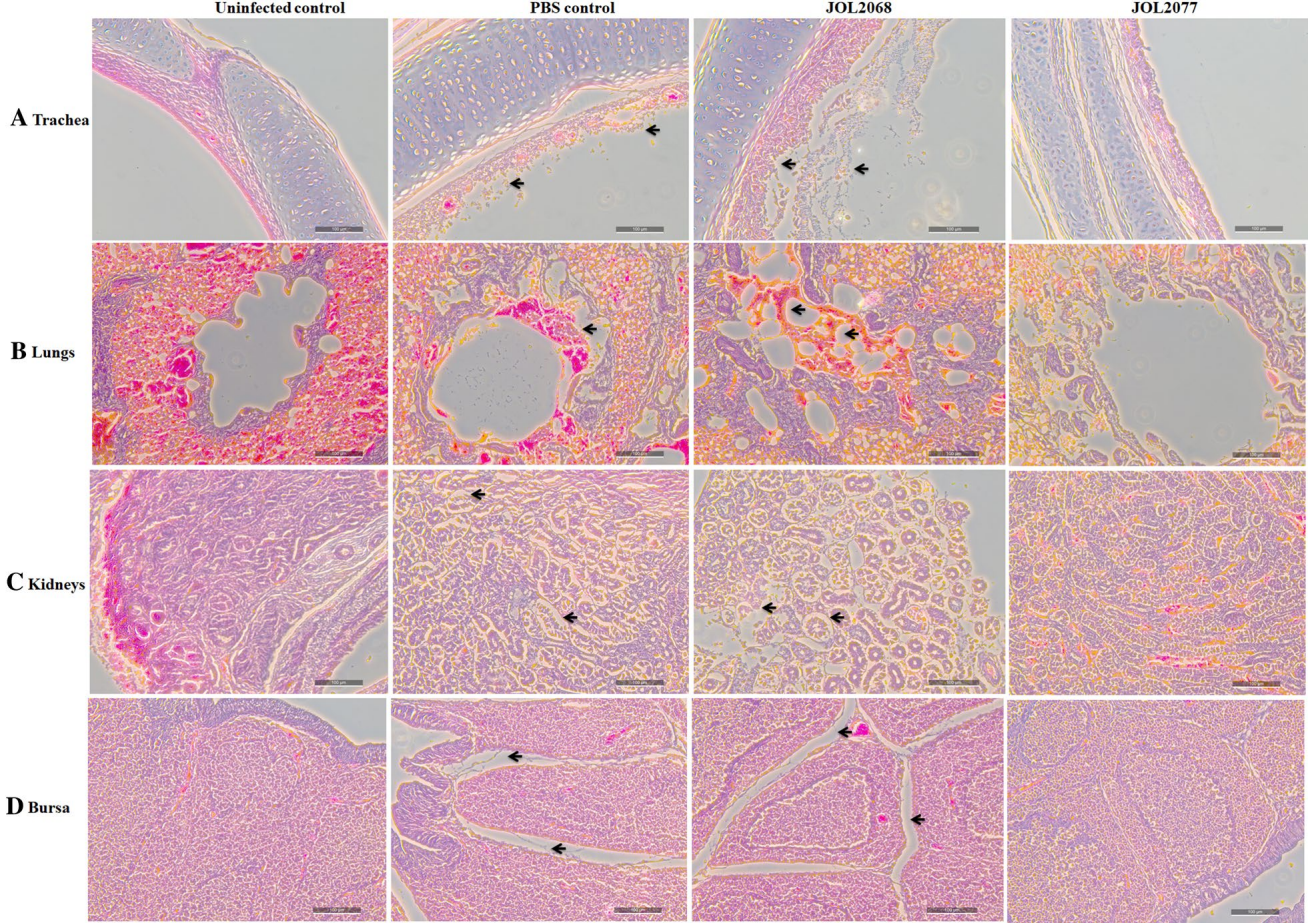
**Photomicrographs of hematoxylin-and eosin-stained lung sections of chickens on 6**^**th**^ **day post-IBV challenge**. Chickens (*N* = 15) were immunized with sterile PBS, JOL2068 or JOL2077, and 28 days later all the vaccinated chickens were challenged with 10^4^ EID50 IBV. At 6^th^ day post-challenge, chickens (*n* = 10) were sacrificed and trachea, lung, kidneys and bursal tissues were collected for histological analysis. Black arrows represent inflammatory lesions.

To further investigate the effect of JOL2077 vaccination on the virus-specific immune protection, histopathological studies were performed on tracheal, lung, kidney and bursal tissues collected from birds on day 6 post-IBV challenge (Figure 4). As expected, no lesions were found in any of the organs collected from the uninfected chickens. In contrast, chickens treated with either PBS or JOL2068 showed significant inflammatory lesions in all of the collected tissues. The trachea of both PBS and JOL2068 chicken groups showed extensive dropout, deciliation, epithelial and glandular desquamation, and infiltration of inflammatory cells (Figure 4A). In lungs, the most prominent histopathological changes observed in PBS and JOL2068 groups were congestion, hemorrhages, and infiltration of lymphocytes into the submucosa of secondary bronchi (Figure 4B). In kidneys, hemorrhages, multifocal necrosis of the renal tubules, and infiltration of mononuclear leukocytes were observed in PBS and JOL2068 control chickens (Figure 4C). The most prominent histopathological changes were observed in bursa of PBS and JOL2068 control chickens. The bursa showed atrophy of lymphoid follicles and widening of the interstitium (Figure 4D). In contrast to JOL2068 and PBS groups, chickens vaccinated with JOL2077 showed insignificant inflammation and the tissues appeared histologically similar to that of normal tissues. Thus, our data clearly indicate that JOL2077 vaccination had offered significant protection against the IBV challenge.

### JOL2077 vaccine induces SG-specific protective immune responses

**Figure 5:**
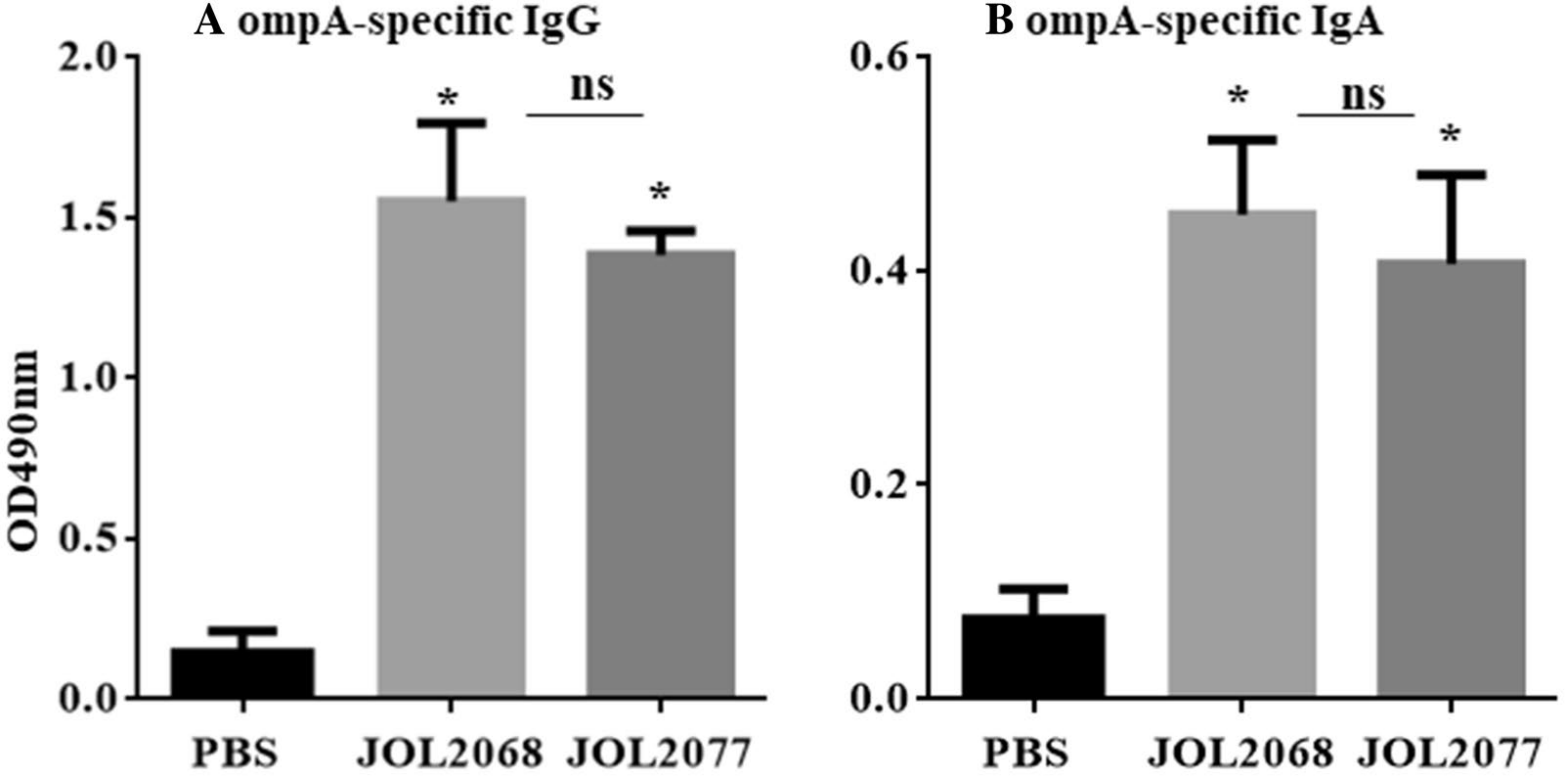
**JOL2077 vaccine induced efficient SG-specific systemic IgG and mucosal IgA responses.** Chickens (*N* = 10) were vaccinated with sterile PBS, JOL2068 or JOL2077, and serum and intestinal wash samples were analysed for ompA SG-specific IgG and sIgA levels, respectively, by an indirect ELISA. **A** Serum IgG. **B** Intestinal sIgA responses. Each data point represents mean ± SD of six chickens. *p < 0.05. ns: non-significant.

To investigate whether JOL2077 vaccine delivering S1 protein can induce SG-specific protective immune responses, three groups of chickens were separately maintained and vaccinated with PBS, JOL2068 or JOL2077, and 4 weeks later SG-specific IgG and sIgA responses were measured in serum and intestinal wash samples, respectively (Figure 5), Our results demonstrated that chickens vaccinated with either JOL2068 or JOL2077 elicited significantly (*p* < 0.05) higher both systemic IgG and sIgA responses compared to the PBS control group. Both JOL2077 and JOL2068 vaccination groups showed comparable levels of antibody responses, suggesting that SG can be exploited to deliver foreign antigens without affecting the SG-specific immunity.

Next, we investigated the protective efficacy of JOL2077 vaccine against FT by orally challenging vaccinated and control chickens with a virulent dose (1.6 × 10^6^ CFU) of wild-type SG bacteria. The mortality of birds, index of protection efficacy in this study, was observed for 12 days post-challenge (Table 3). The chickens vaccinated with either JOL2068 or JOL2077 exhibited lower mortality rate (10%, 1 of 10 birds died) compared to the control chickens, which showed 80% mortality (8 of 10 birds died). The efficacy of JOL2077 vaccine was further evaluated by post-mortem analysis of the survived birds at 12 days post-challenge. Our results showed that the birds vaccinated with either JOL2068 or JOL2077 vaccine had significantly (*p* < 0.05) lower bacterial load in spleen and liver compared to the control group (Table 3).

**Table 3:** **Mortality and bacterial load in the chickens post-challenge**

Groups^a^	Challenge^b^		
	Mortality^c^	Liver^d^	Spleen^d^
PBS	8/10	2/2*	2/2*
JOL2068	1/10	3/9	2/9
JOL2077	1/10	5/9	1/9

^a^7 day old chickens (*n* = 10) were orally immunized with PBS, JOL2068 or JOL2077.

^b^Challenge was performed with a lethal wild-type SG strain (1.6 × 10^6^ CFU) after 28 days post-vaccination.

^c^Number of dead birds upon challenge.

^d^Number of birds positive for SG. *p < 0.05.

## Discussion

This study was aimed to investigate whether attenuated SG delivering S1 protein could induce protective immune responses against both IB and FT in chickens. Our strategy of employing *Salmonella* system as a delivery system for S1 protein has certain advantages over live attenuated or inactivated IB vaccines. These types of vaccines are easy to prepare, stable, convenient, economical, and easy to administer in chickens. Moreover, these types of vaccines does not require exogenous adjuvants as *Salmonella* bacteria provide an appropriate danger signals to the immune system, acting as natural adjuvant, thereby promoting efficient maturation and activation of dendritic cells (DCs) [25, 30], which is prerequisite for the induction of potent adaptive immune responses. Also SG-based IB vaccination can differentiate vaccinated birds from infected ones as only structural and immunogenic antigens are exploited for the vaccine development. Previously, *Salmonella*-based system has been exploited to deliver DNA encoded foreign antigens to induce protective immune responses against a variety of pathogens, especially those invading via the mucosal route [19, 31, 32]. However, a potential limitation associated with the *Salmonella* delivered DNA vaccines is the inability to stably maintain the plasmid DNA inside the bacterium. Moreover, the gene expressions of heterologous antigens encoded by DNA is inefficient, resulting in poor immunogenicity of the vaccine [31], thus, requiring multiple immunizations to elicit some level of significant protection in vaccinated animals [21]. In accordance with this notion, we hypothesized that SG delivering S1 antigen in protein format could induce efficient IBV-specific protective immunity in chickens. We show that single oral inoculation of SG-based S1 vaccine induced efficient protective immune responses against IBV in chickens. We also show that SG delivering S1 protein does not affect the induction of SG-specific humoral and cell-mediated protective immune responses.

The present study demonstrated that orally administered JOL2077 vaccine elicited efficient circulating IgG responses in vaccinated chickens. The role of circulating antibody titers in conferring protection against IBV remains controversial. Some studies have demonstrated that circulating antibodies did not correlate well with the protection from IBV infection [33, 34], while others have shown the importance of humoral immunity in disease recovery and virus clearance [35, 36]. Nevertheless, JOL2077 vaccination had conferred significant protection against IBV challenge and had efficiently contained IBV replication in virus-targeted tissues. Furthermore, JOL2077 vaccinated chickens displayed insignificant inflammatory lesions in IBV-targeted tissues. The role of IgA antibodies in providing resistance to IBV infection is well-documented [35, 37]. The elicitation of IgA antibodies at mucosal surfaces plays an important role in limiting spread of pathogens that primarily enter into systemic circulation via mucosal route. This has direct implication on the pathogenesis and the clinical outcome of the disease. We found significantly higher IgA responses in JOL2077 vaccinated chickens compared to the JOL2068 control group. This finding might explain why JOL2077 vaccinated chickens displayed insignificant inflammatory lesions in targeted tissues and achieved higher level of protection than control groups. Consistent with the notion that cellular immunity is protective against IBV infection [38], we next investigated cytokine and T cell recall responses elicited by our JOL2077 vaccine. After antigenic stimulation, CD4+ T cells secrete anti-viral cytokines, which generate potent antibody formation and CD8+ cytotoxic T lymphocytes, thus playing a critical role in controlling IBV infection [39, 40]. The present study demonstrated that chickens vaccinated with JOL2077 vaccine displayed higher CD4+ and CD8+ T cell responses compared to the control chickens. Consequently, the higher protection rate was observed in chickens vaccinated with JOL2077 vaccine. The nature of the cytokines produced after antigenic stimulation in vitro is an important parameter to define the type of immunity elicited [41]. Our data showed that JOL2077 vaccine induced efficient IBV-specific both Th1 (IFN-γ) and Th2 (IL-4 and IL-10) cytokine responses, which is indicative of the mixed-type of immunity. IFN-γ has potent anti-viral activity through the promotion of natural-killer cells and macrophage activation that are likely to contribute to the containment of IBV replication and spread within the host. Further, IFN-γ upregulates MHC-I and MHC-II molecules and induces IL-12, nitric oxide and superoxide production in macrophages, all of which are important in the elimination of intracellular pathogens [42]. These findings might further explain the higher protection rate observed in JOL2077 vaccinated chickens. The IL-4 and IL-10 have a clear role in promoting humoral immune responses [43, 44]. Previous studies have reported that IL-10 augment the proliferation and plasma differentiation of the B cells that have switched to IgA production, suggesting that IL-10 plays an important role in development of mucosal immunity. Considering that both humoral and cell-mediated immunity are important in containment of IBV replication and clinical disease, our study clearly shows that JOL2077 vaccine is capable of eliciting efficient humoral and CMI responses, and can, thus, offer significant protection against IBV in chickens.

The induction of SG-specific IgG responses following vaccination strongly correlates with the recovery from FT and protection from subsequent challenge infections in chickens [16, 28]. The systemic IgG mediate protection during the early stages of infection when SG circulates extracellularly before it penetrates the target cells [45]. The present study demonstrated that systemic IgG responses were highest in chickens vaccinated with either JOL2068 or JOL2077 strain compared to the PBS control group. Consequently, the mortality rate was lowest in both JOL2068 and JOL2077 vaccinated chickens compared to the chickens that received PBS only. The induction of immune responses at mucosal surfaces serves as the first line defense against pathogens that enter into systemic circulation through mucosal surfaces. Importantly, the presence of antigen-specific secretory (s) IgA antibodies in the intestinal mucus acts as an immunological barrier, thereby preventing the adherence of *Salmonella* bacteria to the intestinal lining and subsequent penetration into deeper tissues [46]. In the present study, we observed that the sIgA levels were highest in JOL2068 and JOL2077 vaccinated chickens. Consequently, the lower bacterial load was observed in spleen and liver of chickens vaccinated with either JOL2068 or JOL2077 strain. These findings clearly suggest that SG can be exploited to deliver viral antigens to elicit carrier and virus-specific humoral and cell mediated protective immune responses.

In conclusion, we show that SG delivering IBV-specific S1 protein can elicit efficient both SG and S1-specific humoral and cell-mediated immunity and can offer dual protection against FT and IB in chickens. Other studies have shown that efficacious live attenuated IB vaccines have protected chickens against clinical signs and mortality and increased significant resistance in birds to infection [40]. Our results clearly show that vaccination with SG-based IB vaccine induced efficient protection against IBV virulent challenge as evidenced by insignificant inflammation in trachea, lungs, kidneys and bursa and by efficient containment of viral replication in tracheal tissues. Further studies are warranted to investigate the protective efficacy of JOL2077 vaccine in both broiler and young layer chickens.

## Additional files

**Additional file 1.** **In silico prediction of linear B cell epitopes and antigenicity of partial S1 amino acid sequence of IBV.** Linear B-cell epitopes and antigenicity of SI protein were predicted by the BepiPred program, which assigns a score to each individual amino acid in a specific sequence. (A) Prediction of linear B cell epitopes. (B) Prediction of antigenicity using Kolaskar & Tongaonkar Antigenicity method.

**Additional file 2.** **Immunoblot analysis of S1 antigen expressed in JOL2077**. The JOL2077 vaccine strain was grown in LB broth to mid-logarithmic phase and then the culture supernatant was collected and subjected to Western blot analysis using polyclonal IBV-specific antibody. Lane M, protein molecular weight (catalog#, P8500, GenDEPOT, USA); lane 1, JOL2077 culture supernatant; lane 2, JOL2068 culture supernatant as negative control.

**Additional file 3.** **Flow cytometric analysis of immune responses** (A) Gating of lymphocytes in flow cytometric analysis of PBMCs isolated from the vaccinated and control chickens. The debris and dead cells were excluded by gating based on the forward and the side scatter. (B) Representative flow cytometry scatter dot plots for CD3+CD4+ and CD3+CD8+ T cell populations of vaccinated and control chicken groups.
