# Supplementary figures and images for: Oral immunization with a novel attenuated Salmonella Gallinarum encoding infectious bronchitis virus spike protein induces protective immune responses against fowl typhoid and infectious bronchitis in chickens
Source: Vet Res. 2018 Sep 12;49:91. doi: 10.1186/s13567-018-0588-9 (PMC6134591; doi:10.1186/s13567-018-0588-9)

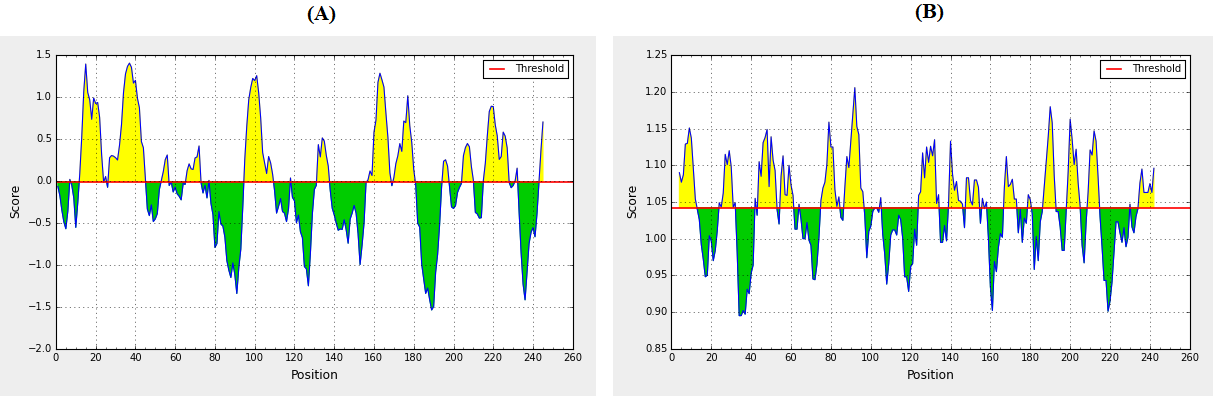

Supplement: Supplementary file 1 — Additional file 1. In silico prediction of linear B cell epitopes and antigenicity of partial S1 amino acid sequence of IBV. Linear B-cell epitopes and antigenicity of SI protein were predicted by the BepiPred program, which assigns a score to each individual amino acid in a specific sequence. (A) Prediction of linear B cell epitopes. (B) Prediction of antigenicity using Kolaskar & Tongaonkar Antigenicity method. [file 13567_2018_588_MOESM1_ESM.tif]

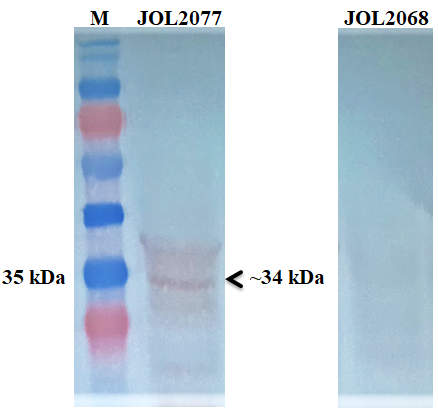

Supplement: Supplementary file 2 — Additional file 2. Immunoblot analysis of S1 antigen expressed in JOL2077. The JOL2077 vaccine strain was grown in LB broth to mid-logarithmic phase and then the culture supernatant was collected and subjected to Western blot analysis using polyclonal IBV-specific antibody. Lane M, protein molecular weight (catalog#, P8500, GenDEPOT, USA); lane 1, JOL2077 culture supernatant; lane 2, JOL2068 culture supernatant as negative control. [file 13567_2018_588_MOESM2_ESM.tif]

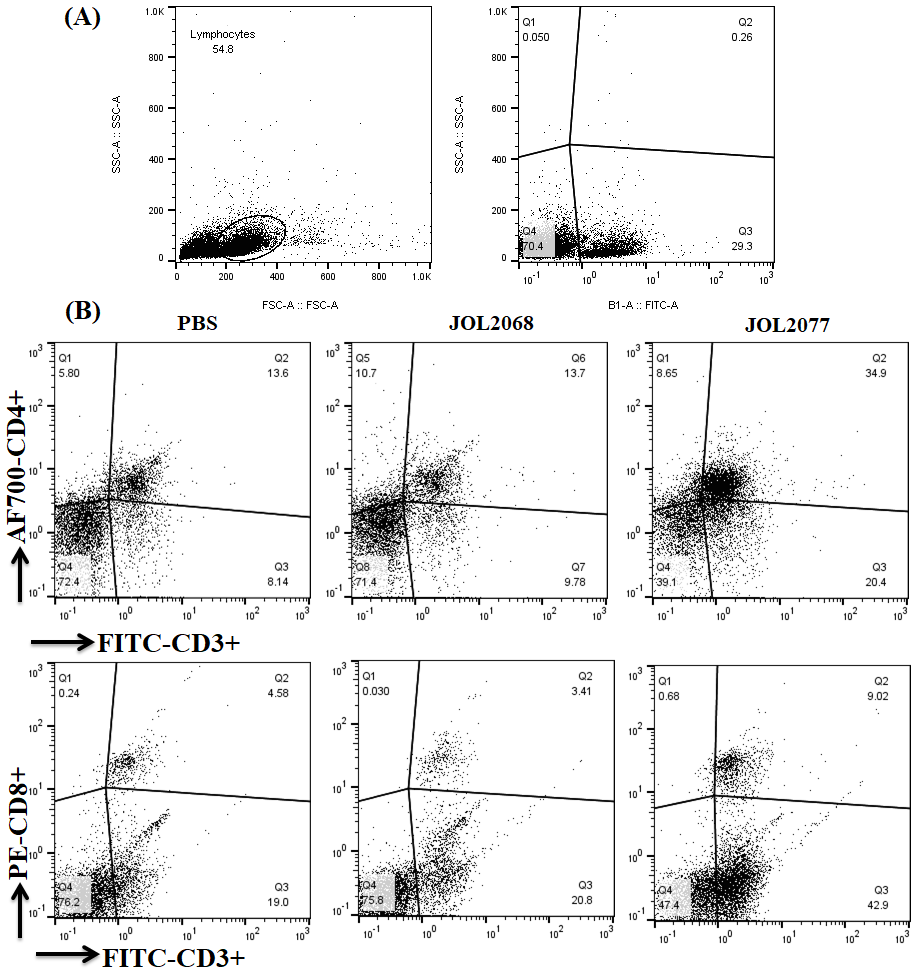

Supplement: Supplementary file 3 — Additional file 3. Flow cytometric analysis of immune responses (A) Gating of lymphocytes in flow cytometric analysis of PBMCs isolated from the vaccinated and control chickens. The debris and dead cells were excluded by gating based on the forward and the side scatter. (B) Representative flow cytometry scatter dot plots for CD3+CD4+ and CD3+CD8+ T cell populations of vaccinated and control chicken groups. [file 13567_2018_588_MOESM3_ESM.tif]
